# Long-term cost-utility analysis of family therapy vs. treatment as usual for young people seen after self-harm

**DOI:** 10.1186/s12962-024-00546-z

**Published:** 2024-05-29

**Authors:** Chris Bojke, David Cottrell, Alex Wright-Hughes, Amanda Farrin, Sandy Tubeuf

**Affiliations:** 1 Leeds Institute of Health Sciences, Academic Unit of Health Economics, University of Leeds, Leeds, UK; 2 Leeds Institute of Health Sciences, University of Leeds, Leeds, UK; 3 Leeds Institute of Clinical Trials, University of Leeds, Leeds, UK; 4 Institute of Health and Society, Institute of Social and Economic Research, Université catholique de Louvain, Louvain-la-Neuve, Belgium

**Keywords:** Cost-effectiveness, Adolescents, QALY, NHS digital, Self-harm, Hospitalisation

## Abstract

**Background:**

The joint evidence of the cost and the effectiveness of family-based therapies is modest.

**Objective:**

To study the cost-effectiveness of family therapy (FT) versus treatment-as-usual (TAU) for young people seen after self-harm combining data from an 18-month trial and hospital records up to 60-month from randomisation.

**Methods:**

We estimate the cost-effectiveness of FT compared to TAU over 5 years using a quasi-Markov state model based on self-harm hospitalisations where probabilities of belonging in a state are directly estimated from hospital data. The primary outcome is quality-adjusted life years (QALY). Cost perspective is NHS and PSS and includes treatment costs, health care use, and hospital attendances whether it is for self-harm or not. Incremental cost-effectiveness ratios are calculated and deterministic and probabilistic sensitivity analyses are conducted.

**Results:**

Both trial arms show a significant decrease in hospitalisations over the 60-month follow-up. In the base case scenario, FT participants incur higher costs (mean +£1,693) and negative incremental QALYs (-0.01) than TAU patients. The associated ICER at 5 years is dominated and the incremental health benefit at the £30,000 per QALY threshold is -0.067. Probabilistic Sensitivity Analysis finds the probability that FT is cost-effective is around 3 − 2% up to a maximum willingness to pay of £50,000 per QALY. This suggest that the extension of the data to 60 months show no difference in effectiveness between treatments.

**Conclusion:**

Whilst extended trial follow-up from routinely collected statistics is useful to improve the modelling of longer-term cost-effectiveness, FT is not cost-effective relative to TAU and dominated in a cost-utility analysis.

## Key points

• Extension of evidence base of hospitalisations from 18 months to 5 years show all hospitalisations and self-harm hospitalisations continue to decline with no incremental difference in performance between family therapy and treatment as usual.

• The conditional cost of a self-harm hospitalisation falls in line with the probability of an hospitalisation. This suggests the constant conditional cost assumed by NICE may be unrealistic. It does not influence the health economic argument in this case but may well do where there is a difference in the rate of hospitalisations across treatments.

• Out of hospital costs represent the main cost-driver and we are unable to estimate the longer-term relationship. Although this is unlikely to have any impact in this evaluation it remains an important research question. Similarly, the relationship between health-related quality of life and self-harm remains uncertain with smaller than anticipated differences measured when using self-harm hospitalisations.

## Introduction

Self-harm, defined as any form of non-fatal self-poisoning or self-injury regardless of motivation or the degree of intention to die, is a major public health issue as demonstrated by the recently updated NICE evidence review on assessing, managing and preventing self-harm [1]. Several studies show increasing trends in the prevalence of self-harm in adolescents [2–5], recent statistics from the Multicentre Study of Self-Harm in England found that 7.3% of girls, and 3.6% of boys, aged 11 to 16, had self-harmed or attempted suicide at some point and the figures were 21.5% for girls and 9.7% for boys between 17 and 19 [6]. Self-harm is associated with an elevated risk of overall mortality [7]; the life years lost to the community due to self-harm, and the impact on family members are significant; recent evidence shows that self-harm led to 320 years of life lost per 100,000 in 2019 in EU [8]. The cost-effectiveness of treatments for self-harming children and adolescents is very sparce and often presents limitations related to small sample of patients, short time horizon, and baseline effectiveness from a single randomised controlled trial [1].

In the context of family-based therapies, several studies have already showed its effectiveness against other types of psychological support or treatment for young people or children for various conditions [9–12]. A number of studies have also investigated the cost of delivering family therapy using administrative records within a large healthcare organisation, insurance claims or Medicaid system and found it less expensive and leading to lower repeated episodes of care [13–16]. However, the joint evidence of the cost and the effectiveness of family-based therapies is limited. A review of the evidence regarding economic studies based on 16 studies concluded that family-based interventions focusing on children and adolescents’ health disorders were cost-effective in only two third of the studies [17]. This review also showed that the quality of the economic evaluations of family-based intervention for young people and children was limited. While a number of studies in North America and Europe simultaneously considered costs and clinical effectiveness or health benefits of family-based therapies to treat young people and children with various conditions, few of them conducted an evaluation considering costs beyond the delivery of the therapy. Only two studies carried out a cost-utility analysis of family-based therapies using EQ-5D to generate quality-adjusted life years (QALYs) including one study using the same trial data as this paper [18, 19] and no prior studies investigated the cost-effectiveness of family-based therapies using a multi-state model in the long-term.

In this paper, we used data from a multi-centre, individually randomised controlled trial comparing family therapy (FT) with treatment as usual (TAU) as an intervention for self-harming adolescents aged 11 to 17 [20]. The within trial economic evaluation found that both trial arms showed an increase in the mean EQ-5D-3L over 18 months follow-up; the largest and significant differences were at 6- and 12-months favouring FT however, there were no significant differences between arms at 18 months. When combined with information on health care resource usage and observed hospital records from NHS Digital (https://digital.nhs.uk) over 18-month follow up as well as the cost of the intervention, the analysis concluded that FT participants incurred £1,266.23 higher costs and gained 0.034 extra QALYs than TAU patients. The incremental cost-effectiveness ratio (ICER) equalled £36,811.80 per QALY indicating that FT was unlikely to be cost-effective as above the NICE cost-effectiveness threshold of £20,000-£30,000.

These 18-month results did not incorporate important uncertainties in the results. Mental health therapies such as family therapies may have an impact beyond the sessions that are attended with teenagers, and their families, reflecting on the therapy after it has finished, and so such interventions may keep on giving and improving individual’s wellbeing beyond the trial follow-up. Adolescents’ cognitive development changes quite a lot over teenage years and beyond since the brain is only considered to be fully developed at the age of 25. It is relevant to look into the change in the long-term as family therapy may require more time to be processed than other therapies but it might also be more sustainable in the long-term. Decision analytic modelling is the preferred method in this context; it usually consists of extrapolating the costs and the consequences by treatment arms using probabilities and assumptions based on the data collected in a trial and data extracted from the literature. The current trial provides us with extended data collection beyond the original 18-month trial follow-up via NHS Digital; this included a longer-term follow-up of self-harm events leading to hospitalisations as well hospitalisations for other reasons during up to 60 months after randomisation. We therefore constructed a longer-term model based on the probability of hospitalisation at each 6 months cycle.

The trial had several unique features. First, the data were collected over 18 months for over 800 adolescents; this sample size was larger than most studies in adolescent self-harm with the smallest study randomising just 39 participants and the largest 448 [21] and the follow up period was longer than many self-harm intervention studies, which typically have a 6-month follow-up [22]. Second, the availability of numerous trial data over four follow-up points (3, 6, 12, 18 months) along with the hospital events, including both A&E and admissions, beyond the trial and up to 36 months after the trial had ended allowed us to consider the relationship between hospitalisation and key variables from the trial. Third, we proposed a quasi-Markov model where we modelled the probability of being in a state using records of hospitalisations across cycles of 6 months. Finally, the extended NHS Digital data also allowed us to test alternative specifications and test the consistency of our results.

## Materials and methods

### The SHIFT trial

SHIFT was a pragmatic, phase III, multi-centre, individually-randomised and controlled trial of 832 adolescents (aged 11–17 years) who had self-harmed at least twice; it was delivered in local child and adolescent mental health services in Yorkshire, Greater Manchester and London between April 2010 and December 2013. Young people were eligible for the trial if they were aged 11–17, living with a primary caregiver, had self-harmed at least twice previously and were referred to child and adolescent mental health services (CAMHS) due to self-harm. Further details of the protocol are reported elsewhere [23]. Following consent and baseline assessment, participants were randomised to receive family therapy plus treatment as usual (hereafter FT) or treatment as usual (TAU) alone. Participants and therapists were aware of treatment allocation however researchers were blind to allocation to ensure unbiased follow-up at 3, 6, 12 and 18-months. Participants were also followed up for hospital attendances using Accident and Emergency (A&E) and in-patient Hospital Episode Statistics (HES) datasets from NHS Digital (https://digital.nhs.uk), augmented by directed hospital record searches [24].

The FT intervention was a modified version of the Leeds Family Therapy & Research Centre Systemic Family Therapy Manual. Qualified family therapists were appointed specifically to work on the trial, received standardised training and worked in teams of 3 or 4, providing trial FT as a team for a cluster of CAMHS. TAU was the care offered to young people by local CAMHS teams and was not restricted in any way. It was expected that TAU would be diverse, involve individual and/or family-orientated work, and be delivered by a range of practitioners.

The original 18-month follow-up was supplemented with extended follow up of a minimum 36 months post-randomisation of hospital records. Longer-term follow-up was available for a sample of 804 (96.6%) of the original sample. The median overall length of follow up was 55.4. The statistical analysis of the clinical outcomes of the extended follow-up has been published elsewhere [25].

### Model overview

A discrete state-based stochastic process model was developed in order to estimate the cost-effectiveness of FT compared with TAU. In line with the within-trial analysis, the base case model adopted an National Health Service (NHS) and Personal Social Services (PSS) perspective and future costs and QALYs were discounted at an annual rate of 3.5% following NICE reference case [26].

#### Horizon

NICE reference case [26] recommends exploring cost-effectiveness over a time horizon that is long enough to reflect any important differences in costs or consequences between treatments being compared. Considering that a cohort of young people will face such a large number of events over their adult life we considered that building a decision analytic model that reflect a lifetime horizon would require a larger number of assumptions with a high level of uncertainty. In line with the recent NICE evidence review [1], we considered that a time horizon of 5 years would be long enough to capture longer-term costs and effects of treatment. The additional advantage here is that we rely on available hospitalisations records and do not extrapolate the course of repeated self-harm.

#### Model states

**Fig. 1:**
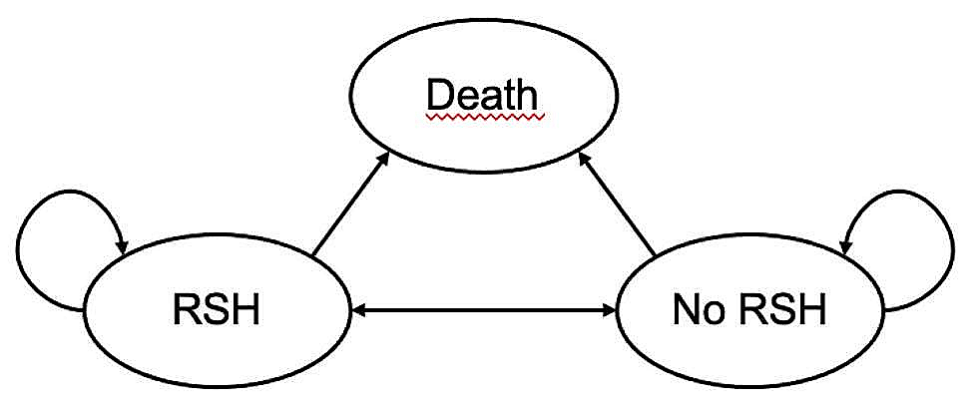
Three-state Markov model

The model structure included three mutually exclusive health states: repeated self-harm (RSH), no self-harming (non-RSH), and death (Fig. 1). Markov models describe patient progression over time through a pathway of health states, with movement between the health states being triggered by events such as hospitalisations or death.

Unlike the standard states-based Markov model where transiting from one health state to the other is based on a transition probability, we directly modelled the probability of being in a state at each cycle estimating a parametric generalized estimating equations (GEE) logistic regression model of the probability of being hospitalised for self-harm at each cycle. The model controlled for patient characteristics such as age group, index hospitalisation cause, gender, and whether the patient was hospital referred, and the interacted effects of a given cycle and the treatment arm. Our model was therefore a quasi-Markov model with health state probabilities estimated directly rather than derived through transition probabilities. In the base case model, repeated self-harming was defined as having been hospitalised for self-harm at least once in a period of 6 months; a 6-month cycle was found to be appropriate to model repeated self-harm [1]. As a secondary analysis, we also considered the probability of any hospitalisation occurring within a given 6 months period as RSH state. Despite the hospitalisation not being coded as self-harm in the HES data, we considered that it would be appropriate to assume that hospitalisations in such a young cohort are rare and when observed, highly likely to be related to self-harm.

There were two deaths over the 60 months follow-up and both deaths were observed in the FT arm between 3 and 4 years post-randomisation. We ran a Kaplan-Meier survival model and found no statistically significant difference between the two arms. The estimated probabilities of death were 0.00289 and 0.00625 for the FT arm at the time of each death while it was 0 throughout for TAU.

Resource use and costs were associated with each health state and patients accumulated costs and health benefits in each state over 6-month cycles.

#### Health data

The primary outcome for the cost-effectiveness analysis was QALYs measured via adolescent’s responses to the EQ-5D-3L at baseline and at 6, 12, and 18 months, converted into health state utility scores using national tariff values and “an area under the curve approach” [27]. Beyond the trial data, we were provided with A&E attendances and inpatient attendances obtained via data downloads from NHS Digital records. We inferred the utilities beyond 18-months using the probability to be hospitalised for self-harm as well as the probability to be hospitalised regardless of self-harm.

We inferred the utilities beyond 18-months first using parametric GEE regression models with normal distribution and linear link function estimating health utility as a function of patient characteristics, self-harm hospitalisation at each 6-month period and treatment * time interactions. We first controlled for the probability to be hospitalised for self-harm and then controlled for the probability of any hospitalisation. As a third alternative, we linked the utilities associated to health states of the economic model (RSH, non-RSH, death) to utility scores as proposed in [1] (Table 1). The utility associated to the non-RSH state corresponds to the EQ-5D-3L derived utility value for adults aged 25–34 years in the general UK population [28] while the utility value for the RSH state was extracted from a secondary analysis using data from this trial [29]. The committee of the NICE evidence review suggested that these values “*were overestimates of the utility relating to each of the two health states, as (…) people who have previously self-harmed (even though they have not self-harmed over the previous 6 months) are unlikely to reach the utility value of the general population, and people who have recently self-harmed (in the last 6 months) are unlikely to have a utility as high*” ( [1] page 67). However, the difference between the two utilities was assumed to be reflective of the true difference between the two health states. These values are assumed to remain constant over time and therefore cost-effectiveness is driven by probabilities in events occurring.

**Table 1:** Parameters from the economic model in the NICE evidence review [1]

Parameter	Value	Distribution	Source
HRQoL Non-RSH state	0.93	Beta (alpha = 2025.242, beta = 152.438	Kind (1999) [28]
HRQoL RSH State	0.68	Beta (alpha = 1529.743, beta = 719.879)	Tubeuf et al. (2019) [29]
Excess Cost RSH State	£2,134	Gamma (alpha = 4, beta = 533.38)	Sinclair (2011) [31]

#### Cost data

Participants’ non-hospital cost data were available at 3, 6, 12 and 18 months in the trial data. It included primary and community care provided by NHS social services, and medications. The cost of the intervention was calculated separately for the FT and TAU arms. It included any treatment details (including duration, number of therapists involved in the session, type, attendance, telephone contact with the family between sessions) as well as the frequency and duration of any supervision meetings recorded. The intervention costs were calculated separately for the FT arm and the TAU arm using a micro-costing approach (see [18] pages 137–138) and were assumed to occur only over the first 12 months for each arm. Resource use and costs were associated with each health state and patients accumulated costs in each state over 6-month cycles as at 3, 6, 12 and 18 months from the trial data. The conversion of resource usage into costs was presented in the main trial paper [20].

Beyond the trial data, we were provided with A&E attendances and inpatient attendances obtained via data downloads from NHS Digital records. We used parametric GEE logistic regression models to infer hospital costs within a given 6-month cycle. The models included estimating (1) hospital costs given a self-harm related hospitalisation has occurred within a given 6-month cycle, (2) hospital costs given any hospitalisation had occurred within a given 6-month cycle, (3) the conditional costs change of a self-harm hospitalisation given a cost for self-harm hospitalisation had occurred within a given 6-month cycle, (4) the conditional costs change of any hospitalisation given a cost for any hospitalisation had occurred within a given 6-month cycle, (5) the probability of an hospitalisation without evidence of SH occurring and (6) the conditional cost when it did. We used a log-link and binary distribution for probability models and Gamma distribution and log-link function for conditional cost models. The matrix of control variables consisted of patient characteristics such as age group, index of hospitalisation cause, gender and whether the patient was hospital referred. The impact of treatment was captured using time * treatment interaction dummy variables. Since the hospital records coincided with model duration there was no requirement for extrapolation and therefore the saturated interaction dummy variable approach allows for maximum flexibility in outcome behaviour over time. We also compared our results with the NICE economic model [1] and considered the excess cost related to the repeated self-harm health state (Table 1). As we did not observe health care costs outside the hospital beyond the trial follow-up, we considered the relationship between hospitalisations and non-hospital costs as observed in the trial to indirectly extrapolate across the post-trial hospitalisation assessment periods.

#### Missing data

We identified two separate problems with missing data. There were observed missing data for EQ-5D-3L and self-reported costs over the trial follow-up (first 18 months) and then missing data for hospitalisations beyond the 18 months due to arbitrary follow-up times. First, we imputed missing EQ-5D-3L, outpatient and other health and social care costs alongside the few missing hospital episode statistics over the first 18 months. We adopted a predictive mean matching to ensure imputed values remained within natural bounds (utilities < 1 and costs ≥ £0). We assumed monotonic missingness and conducted multiple imputations to account for deviations in hospital episode statistics follow-up. Censoring was used to accommodate differences in the length of follow-up. In both cases, we considered 100 imputations.

#### Sensitivity analyses

Deterministic results were combined with Probabilistic Sensitivity Analyses (PSA), which were conducted to assess the impact of parameter uncertainty on the results. Probabilistic analysis consisted of a number of Monte Carlo model simulations with random draws from the regression model estimates and variance covariance matrix that produced different cost and QALY estimates in each simulation of the model. In our model, we chose to do 10,000 simulations. We also conducted several one-way analyses such as removing mortality, changing discount rates, removing non-hospital costs beyond the observed trial, and replacing utilities and SH costs with those preferred by NICE.

## Results

### Descriptive statistics

Table 2 presents the summary of the health care use parameters before and after imputations. The level of imputations was particularly high for the health care costs over the trial data and slightly more marked in the TAU arm. Conversely, hospitalisation data over the long-term had a lower level of missing data especially at early follow-up points, the level of missing data increased over time and was less than 20% in both arms at 42 months follow-up. Missing data in QALY was also important but relatively less marked than for health care costs and FT was also more informed than TAU (Table 3). The within-trial differences in overall costs and QALYs are presented in Table 4. It is important to note that there were no significant differences across treatment arms at any of the within-trial follow-up points neither in costs nor QALYs. The differences in QALY gains favoured FT at 6-months, however gains reduced over time and FT was on average more expensive than TAU at 6- and 18-month follow-up. These differences in costs included any health care use reported as part of the trial follow-up but did not include the intervention costs.

**Table 2:** Observed and imputed health care use parameters per treatment arm within trial and longer-term follow-up

	Family therapy (FT)					Treatment-as-Usual (TAU)				
Time period	*N*	Missing	% Observed	Observed	Imputed	*N*	Missing	% Observed	Observed	Imputed
**Outpatient costs**										
0–3 months	398	182	54.3	£65.34	£63.27	397	205	48.4	£39.43	£49.03
3–6 months	398	200	49.7	£53.79	£76.86	397	245	38.3	£68.38	£74.44
6–12 months	398	166	58.3	£38.16	£50.63	397	219	44.8	£31.72	£43.39
12–18 months	398	203	49.0	£25.66	£25.47	397	244	38.5	£37.74	£41.08
**Health and social care costs**										
0–3 months	398	188	52.8	£279.56	£317.42	397	216	45.6	£394.94	£357.44
3–6 months	398	192	51.8	£301.19	£348.85	397	239	39.8	£365.46	£359.54
6–12 months	398	147	63.1	£567.47	£552.65	397	211	46.9	£615.11	£658.35
12–18 months	398	183	54.0	£536.78	£530.58	397	233	41.3	£723.27	£579.20
**Hospital rates**										
0–6 months	398	3	99.2	37.2%	43.0%	397	5	98.7	35.7%	47.0%
6–12 months	398	3	99.2	28.1%	29.7%	397	7	98.2	30.3%	25.0%
12–18 months	398	6	98.5	24.7%	21.8%	397	12	97.0	24.2%	25.9%
18–24 months	398	14	96.5	21.6%	20.9%	397	16	96.0	19.4%	21.1%
24–30 months	398	17	95.7	15.2%	16.7%	397	19	95.2	18.0%	16.3%
30–36 months	398	18	95.5	11.6%	11.2%	397	22	94.5	13.3%	13.8%
36–42 months	398	52	86.9	12.1%	11.3%	397	53	86.6	10.8%	12.3%
42–48 months	398	100	74.9	10.1%	9.8%	397	111	72.0	8.7%	9.2%
48–54 months	398	174	53.6	8.04%	9.3%	397	179	54.9	10.6%	8.9%
54–60 months	398	239	39.9	5.03%	5.8%	397	247	37.8	7.3%	7.5%

**Table 3:** Observed and imputed mean utility per treatment arm within trial follow-up

	Family therapy (FT)					Treatment-as-Usual (TAU)				
Time period	*N*	Missing	% Observed	Observed mean utility	Imputed mean utility	*N*	Missing	% Observed	Observed mean utility	Imputed mean utility
0–6 months	398	203	49.0	0.7947	0.7736	397	257	35.3	0.7536	0.7382
6–12 months	398	150	62.3	0.8143	0.7975	397	207	47.9	0.7835	0.7925
12–18 months	398	193	51.5	0.8187	0.8139	397	228	42.6	0.8097	0.8133

**Table 4:** Test of differences in health care costs and health utility regressions between treatment arms within trial follow-up (100 imputations)

Parameter	Estimate	Std Error	95% Confidence Limits		Pr > |t|
**Health care costs** - 0–3 Months Diff	-£8.93	£21.26	-£50.61	£32.75	0.67
**Health care costs** - 3–6 months Diff.	£28.70	£40.26	-£50.23	£107.63	0.48
**Health care costs** - 6–12 months Diff.	£5.89	£28.44	-£49.85	£61.64	0.84
**Health care costs** - 12–18 months Diff.	£8.64	£26.48	-£43.27	£60.54	0.74
**Health utility** - 0–6 months Diff.	0.0433	0.023	-0.0017	0.0882	0.06
**Health utility** - 6–12 months Diff.	0.0227	0.0218	-0.0201	0.0655	0.30
**Health utility** - 12–18 months Diff.	0.0076	0.0210	-0.0336	0.0487	0.72

### Estimated hospital costs

**Fig. 2:**
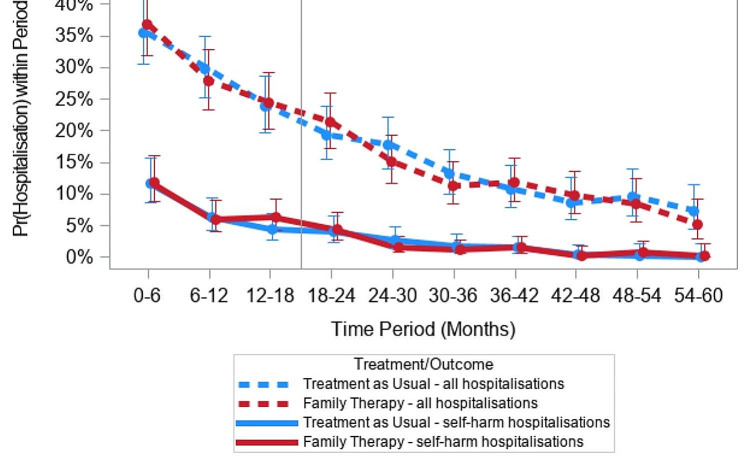
Modelled probabilities of self-harm-related and all hospitalisations over time by treatment arms

The estimated probability of at least one self-harm hospitalisation at each cycle as well as the probability of any hospitalisation at each cycle for each treatment arm are together presented in Fig. 2. There is no incremental difference between the two treatment arms; both FT and TAU observed a diminution of the likelihood of hospitalisations over time. It was however noticeable that both treatment arms show a steady and significant decline in self-harm hospitalisations as well as any hospitalisations over time. The probabilities from these regressions are directly used to predict the shares of the population in the three model health states over time in the absence of transition probabilities being calculated.

The conditional costs of self-harm hospitalisation and any hospitalisation over time and across treatment arms are presented in Table 5 along with the differences between treatment arms and significance levels. The conditional cost of self-harm hospitalisations associated with FT was always higher than with TAU. Significant differences between treatment arms were observed for the periods 0–6 months; 18–24 months and 24–30 months. Beyond 30 months, the point estimates converged in a general downward trend which continued up to 60 months.

**Table 5:** Estimated conditional costs of self-harm hospitalisation and hospitalisation without evidence of SH per treatment arm within trial and longer-term follow-up

	Family therapy (FT)		Treatment-as-Usual (TAU)		Diff. (FT vs. TAU)	
	SH hospitalisation	Hospitalisation w/o evidence of SH	SH hospitalisation	Hospitalisation w/o evidence of SH	SH hospitalisation	Hospitalisation w/o evidence of SH
0–6 months	£1 050.65	£648.29	£684.43	£1076.22	£366.22**	£427.93**
6–12 months	£1 692.41	£940.25	£980.63	£1156.76	£711.78*	£216.51
12–18 months	£1 431.47	£727.97	£667.83	£1204.11	£763.64**	£476.15**
18–24 months	£1 697.69	£437.16	£553.13	£568.07	£1 144.56**	£130.91
24–30 months	£1 042.65	£562.54	£1 042.65	£415.30	£0.00	-£147.24
30–36 months	£657.86	£901.23	£657.86	£328.53	£0.00	-£572.71**
36–42 months	£489.10	£734.25	£489.10	£372.74	£0.00	-£361.52**
42–48 months	£785.75	£440.37	£785.75	£460.23	£0.00	£19.86
48–54 months	£187.67	£416.66	£187.67	£1248.69	£0.00	£832.03**
54–60 months	£146.19	£436.28	£146.19	£252.06	£0.00	-£184.22

Significant levels: **5%, *10%

A similar pattern of results was observed for the conditional costs related to any hospitalisations, with a general downwards trend across both arms and statistically significant differences between treatments particularly over the early period and at 48–54 months with again higher costs in the FT arm. There were also statistically significant differences at 30–36 months and 36–42 months though in this case the TAU arm was associated with higher costs. Given these differences treatment * time interactions were kept at all time periods.

The probability of an hospitalisation without evidence of SH occurring was not related to treatment group but was sensitive to whether a self-harm hospitalisation occurred. We found that for the average participant the probability of an hospitalisation without evidence of SH fell from 34% in months 0–6 to 9% in months 54–60 if a SH hospitalisation occurs in the same time period and from 27 to 6% if hospitalisation without evidence of SH hospitalisation occurred (Table 6). The conditional cost of hospitalisation without evidence of SH was sensitive to both the time period and the treatment arm with FT associated costs being more expensive over the earlier part of the time period (0–24 months) and more mixed afterwards. The cost was not associated with whether an SH hospitalisation had occurred or not. At 0–6 months the average conditional cost was £648 for the FT arm and £1,076 for the TAU arm. At months 54–60 these costs were £436 for FT and £252 for TAU.

**Table 6:** Estimated probability of hospitalisation without evidence of SH conditional on whether SH hospitalisation occurs (treatment invariant)

	SH hospitalisation	Hospitalisation w/o evidence of SH
0–6 months	33.8%	26.8%
6–12 months	29.6%	23.2%
12–18 months	25.5%	19.7%
18–24 months	21.9%	16.7%
24–30 months	18.8%	14.3%
30–36 months	14.6%	10.9%
36–42 months	13.2%	9.8%
42–48 months	12.4%	9.2%
48–54 months	12.2%	9.1%
54–60 months	8.7%	6.4%

### Estimated non hospital costs

We inferred non-hospital costs using self-harm hospitalisations and a GEE regression model and found a significant link between time and the probability of a non-hospital cost occurring with dummy variables for 6-12 months and 12-18 months finding both significant statistical and practical reductions in the likelihood of a non-hospital cost occurring (Table 7). There were no differences between the periods 6–12 months and 12–24 months which suggests the absence of a longer-term trend. A similar argument could be made for SH hospitalisations for the FT arm where there was a flat period between 6 and 12 months and between 12 and 18 months only to be shown as a blip on an otherwise long-term downward trend. There was also a treatment effect between 0 and 6 months but only at that time period whereby the FT arm had a lower probability. A self-harm hospitalisation in the same period led to a higher probability of health care costs outside the hospital. For example, without a self-harm hospitalisation, the probability of health care costs outside the hospital was 96.9% in the TAU arm at 0–6 months and 92.5% for FT. By 12-18 months this probability had fallen to 77.3% for both arms. With a self-harm hospitalisation, the probabilities were 98% for TAU and 96.9% for FT at 0–6 months and 87.9% at 12–18 months for both. In practical terms the increase in probability of health care costs outside the hospital given a self-harm hospitalisation was around 10% points beyond the period 0–6 months.

**Table 7:** Estimated conditional probabilities of non-hospital cost occurring

	Family therapy (FT)			Treatment-as-Usual (TAU)			Diff. (FT vs. TAU)			
	SH hospitalisation	Hospitalisation w/o evidence of SH	SH hospitalisation		Hospitalisation w/o evidence of SH	SH hospitalisation		Hospitalisation w/o evidence of SH		
0–6 months	96.3%	92.5%	98.0%		95.8%	-3.3%**		-1.7%**		
6–12 months	87.2%	76.2%	87.2%		76.2%	0.0%		0.0%		
12–18 months	87.9%	77.3%	87.9%		77.3%	0.0%		0.0%		
18–24 months	87.9%	77.3%	87.9%		77.3%	0.0%		0.0%		
24–30 months	87.9%	77.3%	87.9%		77.3%	0.0%		0.0%		
30–36 months	87.9%	77.3%	87.9%		77.3%	0.0%		0.0%		
36–42 months	87.9%	77.3%	87.9%		77.3%	0.0%		0.0%		
42–48 months	87.9%	77.3%	87.9%		77.3%	0.0%		0.0%		
48–54 months	87.9%	77.3%	87.9%		77.3%	0.0%		0.0%		
54–60 months	87.9%	77.3%	87.9%		77.3%	0.0%		0.0%		

Significant levels: **5%, *10%

The estimated conditional costs of health care costs outside the hospital revealed no relationship between time and treatment variables or any interactions between the two. They were estimated at £705.03 if no self-harm hospitalisation occurred and £933.35 if a self-harm hospitalisation occurred (Table 8).

**Table 8:** Conditional non-hospital costs

	SH Hospitalisation	Hospitalisation w/o evidence of SH
all time points	£933.35	£705.03

### Estimated health utilities

The GEE model estimating health utility showed no statistically significant findings in any combination of treatment and time when accounting for self-harm hospitalisations. This model suggested that the NICE (2022) assumption of time-invariant utility was correct and that differences were driven by episodes of self-harm related hospitalisation. However, whereas two different utility sources were used to estimate a utility difference of 0.25 in [1], our empirical model estimated an expected utility of 0.792 hospitalisation without evidence of self-harm and 0.674 with a self-harm hospitalisation leading to a difference of 0.118, which is almost 50% of the difference imposed in [1] (Table 9).

**Table 9:** Conditional HRQoL Expectations

	SH Hospitalisation	Hospitalisation w/o evidence of SH
All time points	0.674	0.792

### Cost-effectiveness analysis

**Fig. 3:**
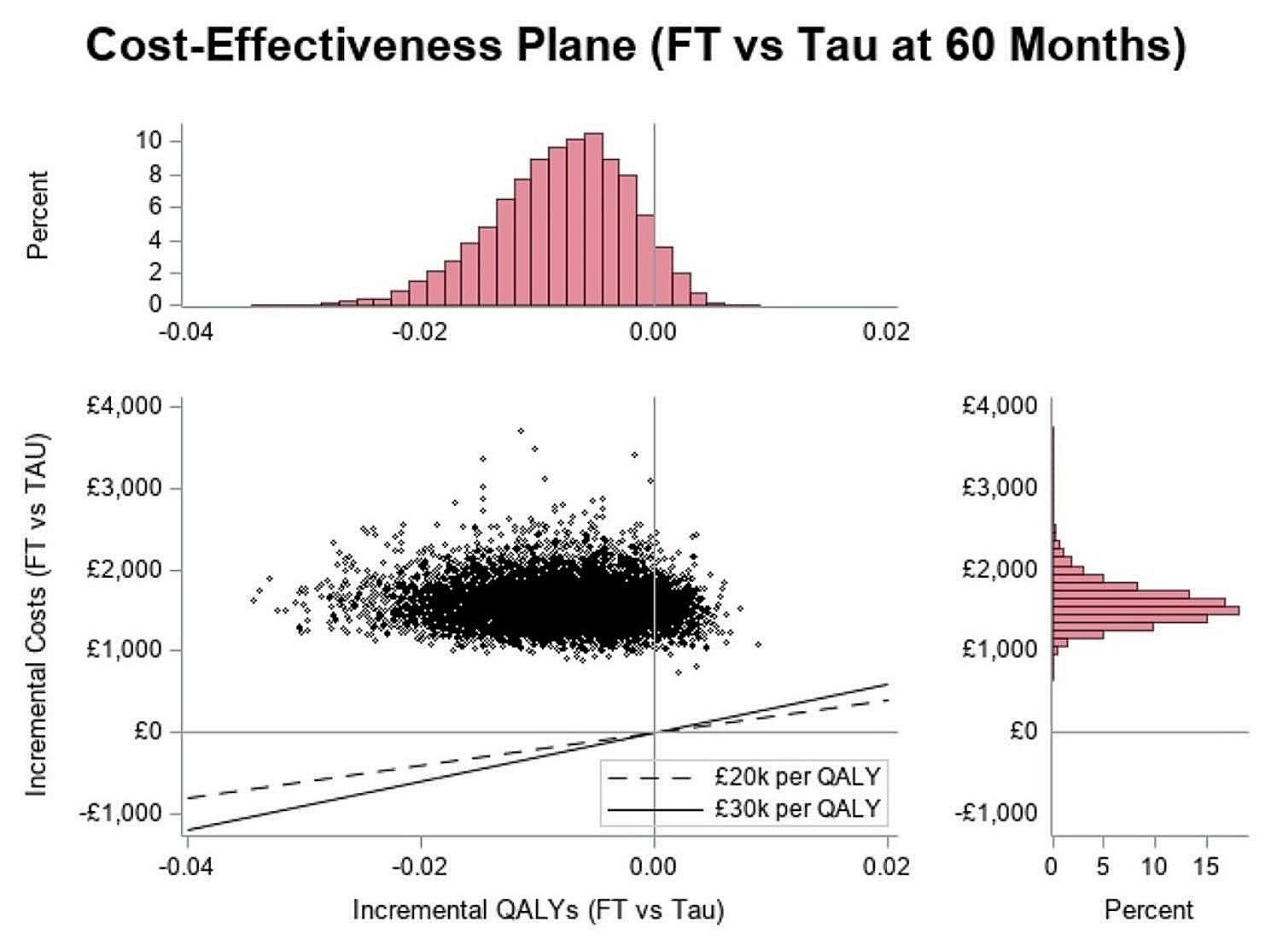
Cost-effectiveness plane of FT compared with TAU (NHS perspective)

**Fig. 4:**
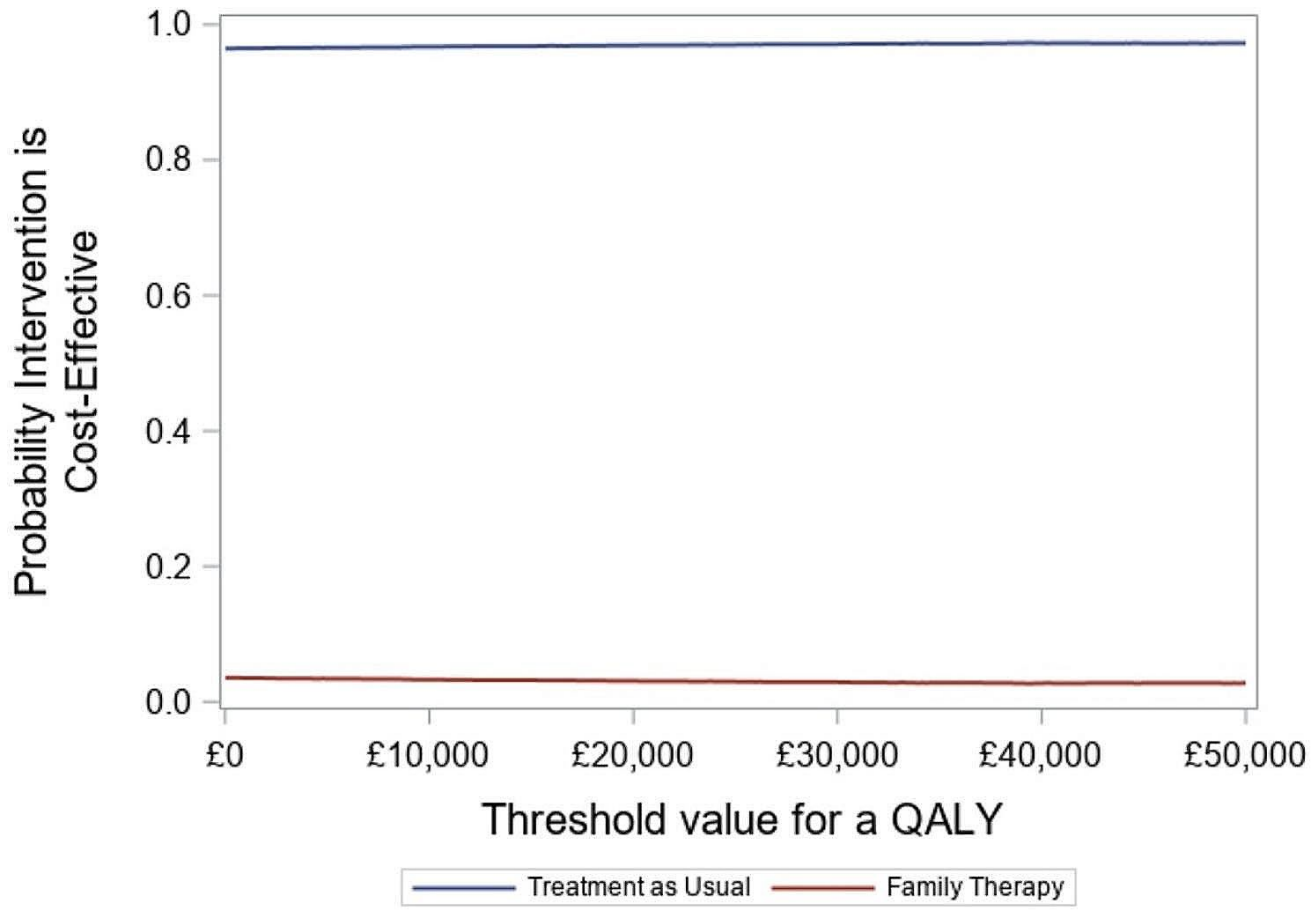
- Cost-effectiveness acceptability curves of FT compared with TAU (NHS perspective)

Results of the deterministic cost-effectiveness analysis are presented in Table 10 and in Fig. 3. FT was associated with an extra cost of £1,693 at 60-month after randomisation and an extra cost of £1,705 at 18-month after randomisation and both decision analytic models exhibited a small negative incremental difference in QALYs (-0.001 and − 0.01). The ICER therefore indicated that FT was dominated (i.e. more expensive and less effective). The points in the cost-effectiveness plane (Fig. 3) were mainly distributed in the north-west quadrant indicating that FT is dominated by TAU over a 5-year horizon. This result differed with the original within-trial analysis, which showed an ICER at 18 months of £36,812 per QALY; this was due to the incremental (but non-significant) difference in self-harm hospitalisations in the 12–18 months period between the two arms. However, given the lack of any significant difference in self-harm hospitalisations anywhere else in the analysis period means FT was likely to be dominated with negative incremental net benefits. The cost-effectiveness acceptability curves are provided in Fig. 4, at 5 years the ICER indicated that FT was dominated and the INHB at £30k was − 0.067.

**Table 10:** Cost-effectiveness results using self-harm hospitalisations as the repeated self-harm state

Original within trial			
**At 18 months**	**Costs (£)**	**QALYs**	
TAU	3,725.49	1.122	
FT	4,991.72	1.157	
	**Incremental cost**	**Incremental QALY**	**ICER (£/QALY)**
FT vs. TAU	1,266.23	0.034	36,811.80

Self-harm hospitalisations as the repeated self-harm state			
**At 18 months**	**Costs (£)**	**QALYs**	
TAU	3,281	1.160	
FT	4,986	1.159	
	**Incremental cost**	**Incremental QALY**	**ICER (£/QALY)**
FT vs. TAU	1,705	-0.001	Dominated
**At 60 months**	**Costs (£)**	**QALYs**	
TAU	7,283	3.68	
FT	8,975	3.67	
	**Incremental cost**	**Incremental QALY**	**ICER (£/QALY)**
FT vs. TAU	1,693	-0.010	Dominated

Self-harm hospitalisations as the repeated self-harm state using NICE cost and utility values			
**At 18 months**	**Costs (£)**	**QALYs**	
TAU	1,268.82	1.349	
FT	2,618.50	1.346	
	**Incremental cost**	**Incremental QALY**	**ICER (£/QALY)**
FT vs. TAU	1,349.68	-0.002	Dominated
**At 60 months**	**Costs (£)**	**QALYs**	
TAU	1,512.79	4.30	
FT	2,845.35	4.29	
	**Incremental cost**	**Incremental QALY**	**ICER (£/QALY)**
FT vs. TAU	1,332.56	-0.001	Dominated

Any hospitalisations as the repeated self-harm state			
**At 18 months**	**Costs (£)**	**QALYs**	
TAU	3,278	1.16	
FT	4,989	1.16	
	**Incremental cost**	**Incremental QALY**	**ICER (£/QALY)**
FT vs. TAU	1,712	0.00004	41,776
**At 60 months**	**Costs (£)**	**QALYs**	
TAU	7,159	3.66	
FT	8,878	3.68	
	**Incremental cost**	**Incremental QALY**	**ICER (£/QALY)**
FT vs. TAU	1,718	0.010	123,153

Using the same model but including the NICE cost and utility values associated with self-harm hospitalisations, the average cost expectations of FT and TAU were much smaller than in the base case. However, the incremental differences remained very similar and led to similar overall conclusions. FT was also found more expensive than TAU and exhibited a small negative incremental difference at 18 months and 60 months follow-up (-0.002 and − 0.001). FT was therefore also found to be dominated by TAU. The incremental net health benefit at £30,000 were − 0.045 and − 0.058 at 18 months and 60 months respectively.

The secondary analysis of the decision analytic model where any hospitalisations were used for the RSH state found slightly different results; FT is associated with a positive incremental QALY at both 18 months and at 60 months compared with TAU. This QALY gain related to the probability of any hospitalisations at 12–18 months being similar between both treatment arms while FT exhibited a higher probability of self-harm hospitalisations at 12–18 months. FT was associated with an extra cost of £1,712 at 18-month and £1,718 at 60-month and small QALY gains (0.00004 and 0.010) compared with TAU, leading FT to being a strategy that is unlikely to be cost-effective as well above the NICE recommended threshold (£20,000-£30,000).

## Discussion

The extended follow-up of hospitalisation up to 60-months from randomisation via the NHS records provided additional data that allowed us to design a decision analytic model where we inferred long-term costs and effectiveness using observed hospital data. We used the probability to be hospitalised for self-harm as well as the probability of any hospitalisation within 6-month cycles and for each arm to infer hospital care related costs as well as associated utilities over the long-term. The extremely low level of missing data in the extended hospitalisation follow-up ensured reduced uncertainty when using multiple imputation techniques. Since self-harm is associated with an acute personal crisis, repetition is likely to reduce over time and this is what we observed over the longer-term with the probability of self-harm hospitalisations as well as any hospitalisations reducing over time. However, when repetition is observed, this suggests more persistent problems associated with a reduced quality of life and additional health care costs. The additional data about expensive events such as hospital admissions, and the advantage of relying on fewer extrapolation assumptions are likely to have led us to provide a more accurate estimate of the incremental costs of FT comparatively to TAU over the 60-months (5 year) time horizon. We found that on the longer-term, FT remained more expensive that TAU with an additional average incremental cost of £1,693. Quality of life was also inferred from the probability to be hospitalised, while hospitalisations events were significantly correlated with reduced quality of life, hospital events are not the only determinants of health utilities. The quasi-Markov model therefore estimated QALY gains with more uncertainty than for costs. The differences in QALYs over the longer-term was estimated close to 0 and often negative. We are therefore confident that FT is dominated in a cost-consequence analysis, which confirms the results of the original cost-effectiveness analysis [19].

Our analysis adds to the recent publication of the economic model of psychological and psychosocial interventions for people who have self-harmed in NICE guidance [1]. Here we found differences in QALYs and costs that were smaller than those used in the recent published NICE guidance and our results also call into question assumptions of invariant time and treatment made in the NICE model especially in the context of extrapolating hospital costs.

The modelling also suggested that lower QALYs and higher costs were associated with self-harm hospitalisations and any hospitalisations, although the differences we found were smaller than the differences used in [1]. We also found that conditional hospital costs were higher for the FT arm over the first 2 years.

Our model was subject to some limitations. First, the analyses considered a patient-level clustering. While a therapist-level clustering could have been relevant, this was not possible as trial recruitment lasted for 36 months and led to many therapists being involved. Second, it was not possible to disentangle whether the observed deaths in FT arm were a chance artefact or a small signal of systematic effect, therefore using the estimated FT death rate is likely to curtail TAU benefit if the impact on mortality is real. Third, we considered that any SH events were allocated to the period in which they occurred, however SH events at each period are unlikely to be independent from SH events observed in other periods. Finally, while the SHIFT trial collected EQ-5D-3L to measure adolescents’ health gains, utility value sets for the youth version of EQ-5D-3L are now available [30] and would be relevant to consider in future trial. Whilst carers’ quality of life was collected as part of the trial follow-up, hospital records were only available for the patients, and it was not possible to extrapolate the health gains to the carers on the longer-term.

This study shows that extended follow-up from routinely collected statistics is useful to improve the modelling of longer-term cost-effectiveness of health care treatments. The high potential of well-informed routinely collected health records is also likely to facilitate a robust estimation of health care related costs; this is extremely useful for policy-makers wishing to make evidence-based decisions in the face of scarce resources.

## Data Availability

Hospital attendance data and costs was derived using data supplied by NHS Digital (DARS-NIC-325074-F0J3D) and are subject to a data sharing agreement between University of Leeds and NHS Digital permitting use of the data for the specific purpose of this project only. Regarding research data, the principal investigator of the SHIFT study has agreed with the NIHR that trial data would not be made freely available in a repository and interested third parties can apply to the trial team for access Clinical Trials Research Unit – University of Leeds – Leeds – LS2 9JT.
